# Research highlight: surgical outcomes of gluteal VY plasty after extensive abdominoperineal resection or total pelvic exenteration

**DOI:** 10.1007/s00423-023-02896-3

**Published:** 2023-04-24

**Authors:** Anke H. C. Gielen, Evie Colier, Shan S. Qiu, Kristien B. M. I. Keymeulen, Laurents P. S. Stassen, Jarno Melenhorst

**Affiliations:** 1 Department of Surgery, Maastricht University Medical Centre, P.O. Box 5800, 6202 AZ Maastricht, the Netherlands; 2 GROW School for Oncology and Reproduction, Maastricht, The Netherlands; 3 Department of Plastic Surgery, Maastricht University Medical Centre, Maastricht, the Netherlands

**Keywords:** Pelvic floor reconstruction, Gluteal VY plasty, Rectal cancer, Extensive abdominoperineal resection

## Abstract

**Objective:**

To describe a suitable alternative technique for reconstruction of the pelvic floor after extensive resection. To review our outcomes of gluteal VY plasty in the reconstruction of the pelvic floor after extensive abdominoperineal resection (conventional or extralevator abdominoperineal resection, total pelvic exenteration, or salvage surgery).

**Design:**

Retrospective cohort study.

**Setting:**

An academic hospital and tertiary referral centre for the treatment of locally advanced or locally recurrent rectal cancer, and salvage surgery in The Netherlands.

**Patients:**

Forty-one consecutive patients who underwent a pelvic floor reconstruction with gluteal VY plasty at Maastricht University Medical Centre between January 2017 and February 2021 were included. The minimum duration of follow-up was 2 years.

**Main outcome measures:**

Perineal herniation is the primary outcome measure. Furthermore, the occurrence of minor and major postoperative complications and long-term outcomes were retrospectively assessed.

**Results:**

Thirty-five patients (85.4%) developed one or more complications of whom twenty-one patients experienced minor complications and fourteen patients developed major complications. Fifty-seven percent of complications was not related to the VY reconstruction. Six patients (14.6%) recovered without any postoperative complications during follow-up. Three patients developed a perineal hernia.

**Conclusions:**

A gluteal VY plasty is a suitable technique for reconstruction of the pelvic floor after extensive perineal resections resulting in a low perineal hernia rate, albeit the complication rate remains high in this challenging group of patients.

## Introduction

Perineal wound problems are a common cause of morbidity after extensive perineal resections [1, 2]. These procedures are most commonly performed in the treatment of locally advanced rectal cancer (LARC). Incidentally, extensive perineal resections are indicated in the surgical treatment of gynaecological malignancy, anal cancer, or severe cases of inflammatory bowel disease (IBD) [3].

Introduction of the extralevator abdominoperineal excision (ELAPE), in combination with increasing use of neoadjuvant radiotherapy in the treatment of rectal cancer, has significantly improved locoregional control of the disease [4, 5]. However, both the use of pre-operative radiotherapy and a larger surgically created pelvic defect further increases this risk of perineal wound healing problems [2, 6]. Wound dehiscence, delayed healing, perineal wound infections, pelvic abscesses, and perineal hernias are the most reported surgical complications [7, 8]. With survival numbers on the rise, treatment-related morbidity has become increasingly important.

In a conventional abdominoperineal resection (cAPR) the total mesorectal excision plane is followed down to the pelvic floor. This leads to a tapering in the resected specimen due to the anatomical narrowing of the distal mesorectum, which may compromise resection margins [4, 9]. The ELAPE procedure entails an *en bloc* resection of the pelvic floor. This results in a cylindrical specimen, with less positive circumferential margins and a lower local recurrence rate than in the cAPR [10–13]. In case of tumour invasion through the mesorectal fascia in other organs, a total pelvic exenteration (TPE) may be necessary to achieve negative resection margins [14–16]. However, the surgically created perineal defect can be challenging to reconstruct.

Currently, there is no consensus on the best surgical technique to close large perineal defects. Primary closure of the perineal wound often results in high tension on the tissues, which increases the risk of dehiscence. Furthermore, it leaves a large dead space that is prone to fluid accumulation and infection [17, 18]. In many cases, primary closure is not possible as the perineal defect after resection is too extensive. Reconstruction of the pelvic floor using a myocutaneous or fasciocutaneous flap reduces the risk of wound complications since the bulk of the flap adequately fills the dead space [19, 20]. Several different techniques have been described to harvest well-vascularized, autologous tissue. The pedicled vertical rectus abdominis (VRAM) flap and the pedicled gracilis flap are the most reported techniques [11, 20–22].

The gluteal VY plasty is preferred in our hospital because of low donor-site morbidity and direct reconstruction of the pelvic floor with vital tissue with intact innervation and vascularization, thus enabling prevention of perineal herniation [22]. The gluteus maximus muscle can be spared during dissection, so its function as a thigh extensor remains intact [23]. The cosmetic outcome of the procedure is generally well accepted by patients since it does not change the seat configuration or silhouette [24].

### Objectives

With this retrospective cohort study, we aim to report on outcomes and technical aspects of gluteal VY plasty in the reconstruction of the pelvic floor after extensive abdominoperineal resection in a tertiary referral centre in The Netherlands.

## Methods

### Setting

In a single-centre observational design, data were retrospectively collected from our hospital’s electronic medical records. Included patients were treated between January 2017 and February 2021.

Every patient enlisted for a TPE, ELAPE, cAPR, or invasive salvage surgery was counselled by a plastic surgeon pre-operatively, in order to explore reconstructive options.

### Patients

All patients who underwent perineal reconstruction by use of a gluteal VY plasty after extensive abdominoperineal resections for both oncological and non-oncological indications were considered eligible for inclusion. The criterion for exclusion was previous perineal reconstructive surgery.

### Surgical techniques

All patients were operated on by one of three experienced colorectal surgeons. An osseous resection of the sacrum and/or coccyx was performed on the indication, if necessary to achieve negative resection margins. The use of an omentoplasty was left to the discretion of the surgeon. All perineal reconstructions were performed by a senior staff member of the department of plastic surgery. The surgical technique used to harvest the VY plasty is based on the technique described by Hainsworth et al. in 2012 [25].

**Fig. 1:**
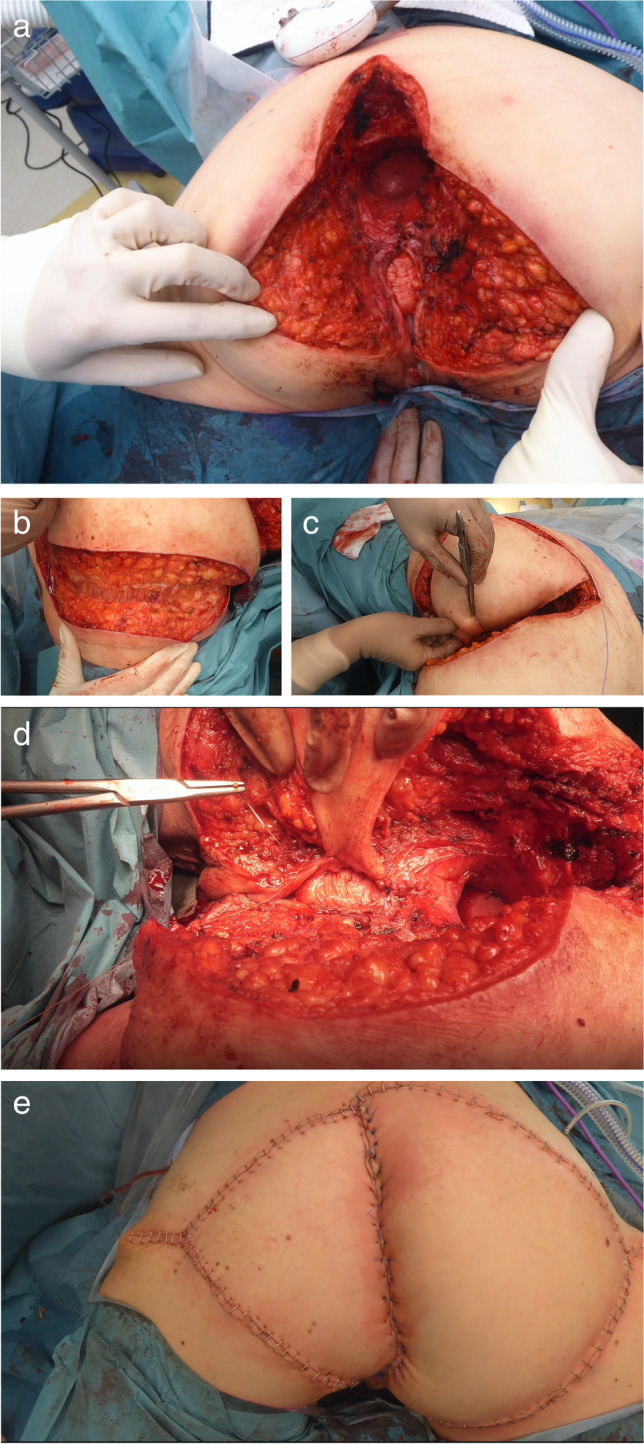
**a** The perineal defect. **b** Incision down to the fascia. **c** Mobilizing tissue in medial direction. **d** Vaginal reconstruction. **e** Direct postoperative results

Based on the extent of the defect caused by the pelvic resection (Fig. 1a) and the impairment of the pelvic floor, a unilateral or bilateral VY plasty of the gluteal area was harvested by the plastic surgeon. Skin markings were drawn after the completion of the pelvic resection with the patient in the prone position. Skin and fasciocutaneous tissue were then incised down to the gluteus maximus muscle fascia (Fig. 1b). Vascularization and innervation remain intact, as the major vascular input is derived from perforators of the inferior gluteal artery. The tissue is mobilized medially (Fig. 1c) until the flap can be sutured to the sacrotuberous ligaments to recreate the pelvic floor and, if necessary, also the posterior wall of the vagina (Fig. 1d). The excess skin on the medial side of the flap is de-epithelialized. This part of the flap is flexed inward to fill the defect. One drain is placed routinely on the abdominal side of the pelvic field. It is left to the discretion of the plastic surgeon to place a second, more superficial drain along the flap in the subcutaneous tissue. The skin is closed per layer with interrupted absorbable sutures and surgical staples (Fig. 1e). Drains are kept in place for a minimal duration of seven days and are removed once the output is less than 30mls in 24 h. Post-operatively, patients were strictly confined to a specific bed with Air Fluidised Therapy (AFP) for fourteen days to reduce pressure on the flap and facilitate wound healing. AFP creates optimal micro-climate control with minimization of friction and pressure on large perineal wounds [26]. A physical therapist looked after adequate mobilization of the extremities during this fortnight. The postoperative complications in perineal wound healing were retrieved from patient records and graded according to the Clavien-Dindo classification [27]. The Comprehensive Complication Index ® (CCI) was computed using the CCI calculator [28, 29].

### Follow-up

After discharge from the hospital, all patients were seen in the outpatient clinic approximately six weeks after the surgery. Oncological follow-up was performed in our surgical outpatient clinic according to the Dutch guideline for the treatment of rectal cancer in all patients with LARC or locally recurrent rectal cancer (LRRC) [30]. The minimal follow-up time was 2 years.

### Endpoints

The primary outcome was the occurrence of perineal herniation. Perineal herniation was defined as a perineal bulging mass, associated with pain and discomfort, as diagnosed in patient history and physical examination. If there was any uncertainty about the clinical presentation, an abdominal computed tomography (CT) scan would be considered to confirm the diagnosis.

Secondary outcome measures were perineal wound healing and other complications, graded according to the Clavien-Dindo classification. Uncomplicated healing was defined as a normal postoperative course without the need for pharmacological treatment or any intervention [27]. Minor wound complications were defined as a Clavien-Dindo Grade I or II and included perineal wound dehiscence or perineal wound infection and partial flap necrosis. Wound dehiscence is defined as the splitting open of a previously closed surgical incision site [31]. This dehiscence might be caused by an underlying surgical site infection but does not necessarily have to be.

Major wound complications were defined as a Clavien-Dindo grades III–V and included presacral abscesses, wound-related readmissions to the hospital, and perineal hernia. All further complications until 30 days postoperatively were registered. Additional tertiary outcomes were the length of hospital stay and disease-free survival.

## Results

The population consisted of 25 (61%) male and 16 (39%) female patients with a mean age of 63.2 (45–82) years (Table 1). Twenty-nine patients underwent an oncological resection (nineteen for LARC, eight for LRRC, one for recurrent anal carcinoma, and one for recurrent squamous cell carcinoma of the vagina). Twelve patients were operated on for benign indications. Partial sacrectomy was indicated in twenty-three patients. Twenty-one patients underwent a total pelvic exenteration. Nine patients underwent an ELAPE. A conventional APR was performed on two patients. In four, extensive resection of the fistulous complex including the anus and rectum was performed. Two procedures were the extensive resection and debridement of a large non-healing perineal wound after a previous ELAPE, two of an epithelialized sinus after a previous ELAPE, and one for a recurrent presacral abscess after a previous low-anterior resection (Table 2). Twenty-four patients (58.5%) received a simultaneous omentoplasty. In one particularly challenging case (in which a LARC combined with a complex fistula in Crohn’s disease was the indication for pelvic exenteration), the large perineal defect required the addition of a Permacol™ mesh to the gluteal VY plasty in order to recreate the pelvic floor.

**Table 1:** Patient characteristics (*N* = 41)

Age	63.2 (40–82)
Male	25 (61.0%)
Female	16 (39.0%)
BMI	25.53 (16.79–38.06)
Active smoking	
Yes	8 (19.5%)
No	33 (80.5%)
Diabetes mellitus	
Type I	1 (2.4%)
Type II	5 (12.2%)
No	35 (85.4)
Malignancy in history	
Yes	29 (70.7%)
No	12 (29.3%)
Inflammatory bowel disease	
Crohn’s disease	2 (4.9%)
Ulcerative colitis	4 (9.8%)
No	35 (85.4%)
Operation indication	
LARC	19 (46.3%)
LRRC	8 (19.5%)
Complex perianal sinus and fistulas	5 (12.2%)
Other*	9 (22.0%)
Preoperative radiotherapy	
Yes	26 (63.4%)
No	15 (36.6%)
Previous perineal radiotherapy	9 (22.0%)
Preoperative chemotherapy	
Yes	28 (68.3%)
No	13 (31.7%)

*Age in years; mean, BMI: Body Mass Index in kg/m*^*2*^*; mean*

*LARC* locally advanced rectal cancer, *LRRC* locally recurrent rectal cancer

^*^ Other operation indications were; a non-healing perineal wound, recurrent presacral abscess, recurrent anal carcinoma, recurrent squamous cell carcinoma of the vagina, and persistent pain due to a rectal prolapse after anal cancer

**Table 2:** Surgical details (*N* = 41)

Surgical procedure	
cAPR	2 (4.9%)
ELAPE	9 (22.0%)
Robot-assisted	4 (9.8%)
TPE	21 (51.2%)
Excision chronic sinus	4 (9.8%)
Other°	5 (12.2%)
Osseous resection	
Yes	23 (56.1%)
No	18 (43.9%)
Gluteal VY plasty	
Unilateral right	6 (14.6%)
Unilateral left	10 (24.4%)
Bilateral	25 (61%)
Omentoplasty	
Yes	24 (58.5%)
No	17 (41.5%)
Vaginal reconstruction	
Yes	5 (12.2%)
No	36 (87.8%)
Postoperative stay (d), (range)	22.0 (9—55)

*cAPR* conventional abdominoperineal resection, *ELAPE* extra-levator abdominoperineal excision, *TPE* total pelvic exenteration*, Postoperative stay in days: mean*

° Other surgical procedures were: extensive resection and debridement of an extensive non-healing perineal wound, epithelialized sinus, or presacral abscess

Twenty-five patients underwent a perineal reconstruction with a bilateral gluteal VY plasty. Sixteen patients received a unilateral gluteal VY plasty. One patient needed an additional advancement flap in order to cover the entire pelvic defect with viable tissue. The overall median hospital stay was 22 days (range 9–55 days). One patient was permitted to discontinue treatment in the AFP bed after seven days.

Three patients developed a perineal hernia: two of these patients were 1.5 years after the surgery, and the third patient was already two years post-operative. The occurrence of postoperative complications is shown in Table 3. Six patients (14.6%) have recovered without any postoperative complications. Twenty-one patients (51.2%) have encountered minor (wound) complications, and fourteen patients (34.1%) suffered from major complications. No complete flap necrosis was seen, and no perineal re-operations were performed. All cases of wound dehiscence and partial flap necrosis could be managed with conservative treatment (Table 4).

**Table 3:** Postoperative complications

Complications*	
No complications	6 (14.6%)
*Gluteal VY plasty*	
Perineal wound dehiscence	11 (26.8%)
Perineal wound infection	3 (7.3%)
Partial flap necrosis	3 (7.3%)
Perineal abscess	1 (2.4%)
*Intra-abdominal*	
Presacral abscess	5 (12.2%)
Urinary tract infection	10 (24.4%)
Urosepsis	2 (4.9%)
Urinary retention	4 (9.8%)
Ileus	9 (22.0%)
Relaparotomy	3 (7.3%)
*General*	
Pulmonary embolism	2 (4.9%)
Other^†^	10 (24.4%)
Complications at follow-up	
Perineal hernia	3 (7.3%)
Readmission to hospital	5 (12.2%)
Recurrence of fistula	1 (2.4%)

^*^ Complications occurred during hospitalization

^†^Other reported complications were: urinoma, seroma, atrial fibrillation, hypokalaemia, facial oedema, prolonged need for post-operative vasopressor infusions, iatrogenic pancreatic injury, Transient Ischemic Attack of the left hemisphere, and hypernatremia

We did not find any difference in the occurrence of total complications or wound complications between the patients with or without active smoking or diabetes. We did not observe a correlation between BMI and complications. The six patients who recovered without any complications had an average body mass index (BMI) of 22.71, and none of them suffered from diabetes. Three out of the fourteen patients (21.4%) who developed major complications suffered from diabetes. We could not find any difference in the use of an omentoplasty on the development of a perineal hernia. Of the three patients with a perineal hernia, two patients received an omentoplasty, one patient did not. Two of these three patients also experienced minor complications in wound healing, one patient developed dehiscence, and the second patient experienced a superficial perineal wound infection. The third patient did not experience any perineal wound healing problems.

**Table 4:** Complication classification (*N* = 41)

Clavien-Dindo classification	
No complication	6 (14.6%)
Grade I	6 (14.6%)
Grade II	15 (36.6%)
Grade IIIa	6 (14.6%)
Grade IIIb	3 (7.3%)
Grade IV	5 (12.2%)
Grade IVa	0
Grade IVb	0
Grade V	0
CCI (range)	26.91 (0–58.7)

*CCI* comprehensive complication index, average

Eight patients died during follow-up after surgery. Distant metastases were the underlying cause of death in five of these patients. One patient died from a local recurrence of malignancy, one from intestinal ischemia in an obstructive ileus due to adhesions two years after surgery, and one from a myocardial infarction after knee surgery.

## Discussion

Forty-one consecutive patients underwent a gluteal VY plasty for the reconstruction of the pelvic floor after extensive abdominoperineal resection. This study shows similar numbers on incidence of both perineal herniation and perineal wound healing problems as previous studies on different surgical techniques for closure of large perineal defects [2, 6, 9]. Thereby demonstrating that a gluteal VY plasty can be considered a suitable alternative technique.

Several techniques for perineal reconstruction with myocutaneous flaps have been introduced in order to prevent a perineal hernia. The review of Foster et al. [32] reported widely varying incidences of perineal herniation. However, there was consensus on the fact that primary closure resulted in the highest risk of perineal herniation with an incidence of 26%. Perineal herniation after the closure of the defect with the use of biosynthetic mesh varied from 0 to 14%, the reported incidence after myocutaneous flap closure is found to vary from 1 to 11%. The pedicled Vertical Rectus Abdominis Myocutaneous (VRAM) flap is amongst the most reported techniques. The fact that this tissue has not suffered any previous radiation damage, makes this well-vascularized muscle a good candidate to fill the large defect in the pelvic cavity. One would expect fewer problems in perineal wound healing in the short term, and preventing herniation in the longer term. The fact that the flap can be harvested through the already-created median laparotomy wound provides another advantage [19, 22]. A disadvantage is the fact that the harvesting of the rectus abdominis might interfere with the placement of the stoma. Furthermore, there have been consistent reports of weakness in the abdominal wall at the donor site, resulting in abdominal wall herniation and requesting an operative correction. This weakness can also interfere with the function of a necessary stoma. Also, the fact that the rectus abdominis muscle is denervated makes this particular myocutaneous flap prone to the risk of loss of volume over time, which might actually further increase the risk of perineal herniation [10, 21, 22]. This is also demonstrated by the review of Radwan and colleagues. They describe a complete flap loss rate of 1.8% in a total of 1827 included patients, with a mean donor site morbidity of 15% [33].

The use of the pedicled gracilis muscle for a myocutaneous flap avoids the abdominal donor-site morbidity of the VRAM flap. It provides a good alternative method for perineal reconstruction, especially in the case of laparoscopic resection and the need for the placement of a stoma [34]. However, the rather lean gracilis muscle provides only a limited bulk of muscle tissue, often requiring a bilateral procedure in an attempt to fill the pelvic defect [21]. Furthermore, the arc of rotation of the gracilis muscle may not be sufficient to reach the most cranial point of the perineal defect. As concluded by Musters and colleagues in 2014, neoadjuvant radiotherapy is the determining factor that increases perineal wound problems after an APR. They also suggest that the extent of the resection is not the most dominant factor [9]. Our finding that ten out of eleven patients who developed major wound complications had been treated with at least one course of radiotherapy (either directly prior to the pelvic resection in the treatment of LARC or LRRC, or earlier for an unrelated indication such as prostate cancer) is in line with this conclusion.

Factors such as smoking, diabetes, and body mass index (BMI) have been suggested to increase the risk of both minor and major complications in perineal wound healing [3, 35, 36]. We could not confirm this finding in our study, but this may be due to the limited number of patients.

Perineal wound problems after primary closure of the pelvic defect have also been attributed to the created dead space in the pelvis, which is prone to fluid accumulation and infection [17, 18]. The use of an omentoplasty has been suggested to decrease this risk since it fills the large cavity with viable tissue [6, 37]. However, Blok and colleagues concluded in 2018 that an omentoplasty after an APR with primary closure of the pelvic defect did not only fail to improve perineal wound healing but also tended to increase the occurrence of perineal hernias [38]. The use of an omentoplasty could be more promising in combination with a myocutaneous flap rather than as a stand-alone solution. Numbers in this cohort are too small for a definite conclusion on this topic.

### Interpretation

The gluteal VY plasty has not yet been widely used in the perineal reconstruction. This might be caused by the fact that the tissue is thought to have been exposed to previous radiation therapy. Furthermore, the current protocol of fourteen days with AFP is a real challenge for our patients and we are looking for ways to shorten this time in order to reduce the length of hospital stay and treatment costs as well while maintaining the beneficial effects on wound healing. However, an advantage of this local flap to a distant one is the prevention of additional donor site morbidity [24]. The intact abdominal wall prevents abdominal weakness and avoids interference with stoma placement [39]. The fact that the flap remains innervated and well-vascularized makes it less prone to volume loss over time. The robust vascularization reduces the risk of flap failure to a minimum. The gluteal VY plasty is a versatile advancement flap that can also be used in partial vaginal wall reconstructions [25, 40]. The role of synthetic or biological mesh in the prevention of perineal herniation is still highly debated. A single-centre randomized controlled trial is being conducted at the moment in order to compare the results of a porcine biological mesh and gluteus maximus myocutaneous flap closure of the perineal wound after ELAPE: the NEAPE trial [41].

### Limitations

An important limitation of this retrospective cohort study is the fact that this cohort of 41 patients—although large in its kind—is too small for relevant statistical analysis. Furthermore, a direct comparison between the different reconstructive techniques is rather difficult since no comparative studies have yet been performed on the matter.

## Conclusion

Gluteal VY plasty is a suitable technique for the reconstruction of the pelvic floor after extensive pelvic resections. The occurrence of a perineal hernia can be prevented in the majority of patients since the VY plasty adequately fills the dead space created upon resection. In general, we believe that the use of myocutaneous or fasciocutaneous flaps contributes to a significant reduction of complications. Although also with a gluteal VY plasty, complications in perineal wound healing cannot be abandoned, and evidence to choose one method over the other is scarce. The gluteal VY plasty offers a viable alternative with good local results and the absence of distant donor-site morbidity.

## Data Availability

Data available on request from the authors.

## Declarations

### Preregistration

No preregistration exists for the reported studies reported in this article.

The authors report no specific funds or grants for this study. All authors contributed to the study conception and design. All authors read and approved the final manuscript.

Approval of the Ethics Committee was not necessary for this retrospective cohort study.
